# Utility of PLUS CYCLE to measure physical activity and sleep duration and detect postoperative sleep disturbances in hospitalized dogs

**DOI:** 10.1371/journal.pone.0318475

**Published:** 2025-06-26

**Authors:** Akihiro Ohnishi, Makoto Yamamoto, Natsuki Akashi, Eri Iwata, Taketoshi Asanuma, Yoshiki Itoh

**Affiliations:** 1 Department of Veterinary Medicine, Faculty of Veterinary Medicine, Okayama University of Science, Imabari, Japan; 2 Japan Animal Referral Medical Center, Inc., Kawasaki, Kanagawa, Japan; Ehime University Graduate School of Medicine, JAPAN

## Abstract

Sleep is essential for animal health and welfare. In humans, postsurgical sleep disturbances can delay postoperative recovery. However, objective sleep evaluation in dogs and studies of postoperative sleep disturbances in animals have not been reported. This study aimed to determine whether activity monitors (PLUS CYCLE^®^; JARMeC, Kanagawa, Japan) can accurately monitor the condition of hospitalized dogs and postoperative sleep disturbances. First, the activity data collected by PLUS CYCLE^®^ were compared with the observation data in a video of hospitalized dogs (n = 9). We determined the correlation between the total hours of physical activity, Sleeping/Resting time and amount of physical activity collected by PLUS CYCLE^®^, and the time when body movements could not be confirmed (inactive time) recorded by video. There was a strong correlation between the inactive time observed in the videos and the Sleeping/Resting time in PLUS CYCLE^®^ (p < 0.0001, r = 0.95). Thereafter, six hospitalized dogs (n = 6) that underwent phacoemulsification and aspiration surgery for cataracts at the Veterinary Teaching Hospital of the Okayama University of Science were monitored to compare pre- and postoperative amounts of physical activity and Sleeping/Resting time between two groups: the Colorado State University Acute Pain Scale (APS) = 0 group (n = 3) and the APS = 1 group (n = 3). A significant decrease in postoperative sleep duration was observed in the APS = 1 group (p = 0.0224). This prospective study suggested that PLUS CYCLE^®^ can accurately assess the condition of hospitalized dogs and that it has the potential to detect postoperative sleep disturbance. Thus, PLUS CYCLE^®^ may help manage postoperative hospitalization and pain management.

## Introduction

Sleep is important for health in animals and is necessary for body repair and homeostasis [1], learning and memory consolidation, and immune system function [2]. Proper sleep is essential for the welfare of animals. Previous reports have examined the correlation between sleep patterns and the welfare status of dogs [3]. While sleep can be investigated in humans through questionnaires [4], it is challenging to assess animal sleep. It is difficult for dog owners to make accurate judgments of their dog’s sleeping pattern, as they are usually unable to watch their animals closely throughout the night. In addition, studies on sleep in dogs and cats are limited.

Knazovicky et al. [5] used the Sleep and Night Time Restlessness Evaluation (SNoRE) score to assess sleep quality in dogs and reported an association between chronic pain in arthritis and sleep quality. However, the SNoRE score is a subjective evaluation by the owner and does not lead to an objective evaluation. Polysomnography (PSG) is an objective assessment [6], but it is not easy to use in clinical practice and is unsuitable for routine sleep assessment.

In human medicine, postsurgical sleep disturbances can delay postoperative recovery, which is influenced by the environment in which the patient is hospitalized and other factors [7]. Animals are not accustomed to the environment of veterinary hospitals and are surrounded by stressors [8]. Studies on sleep conditions in hospitalized animals and postoperative sleep disturbances in animals have not yet been reported.

In recent years, the use of activity monitors, such as accelerometers, has been widely reported in humans, with some studies comparing self-reports and activity monitors for physical activity [9] and the detection of deterioration in physical function [10,11]. Additionally, methods to assess sleep using activity monitors and their usefulness in monitoring neurological diseases in humans have been reported [12].

PSG, which includes EEG monitoring, is considered the gold standard for sleep assessment. However, the invasive nature of PSG, especially in veterinary settings, limits its use in clinical practice. Therefore, this study employed the PLUS CYCLE® system as a non-invasive alternative to estimate rest and sleep duration.

The PLUS CYCLE^®^ is a non-invasive activity monitor explicitly developed to measure the activity of dogs and cats, with a built-in three-directional accelerometer and air pressure sensor. The PLUS CYCLE^®^ has a diameter of 27 mm, thickness of 9.1 mm, and weighs 9 g and is connected and paired to a smartphone via Bluetooth to access the activity data. The PLUS CYCLE^®^ allowed for the accurate assessment of activity and sleep duration in cats [13].

This study aimed to determine whether PLUS CYCLE^®^ can be used to accurately monitor the condition of hospitalized dogs and whether dogs, like humans, experience postoperative sleep disturbances.

## Materials and methods

Canine patients who underwent ophthalmic surgery at the Veterinary Education Hospital of Okayama University of Science between April 2020 and June 2021 were included in the present study. Dogs that required nighttime care owing to concurrent diseases, those given analgesics after 18:00, and those whose surgery was completed after 18:00 were excluded. This study was approved by the clinical research and trial ethics committee, Veterinary Education Hospital, Okayama University of Science (2021−0009). All owners of the dogs that participated in the present study gave written consent for data collection.

### Experiment 1: A comparison of activity data collected PLUS CYCLE^®^ and video observation of hospitalized dogs

Nine dogs were recruited for Experiment 1, with mean age and body weight of 9.3 ± 2.9 years (range, 5.5–13.9 years) and 6.6 ± 3.0 kg (range, 2.7–11 kg), respectively. There were two unneutered males, and two unspayed females, one neutered male and four spayed females. The breeds included Toy Poodle (n = 3), Shiba Inu (n = 2), and Miniature Dax, Miniature Pincher, Bichon Frise, and mixed breed (n = 1 each) (Table 1). All dogs included in the study were non-brachycephalic breeds. PLUS CYCLE^®^ was attached to the neck collars of the subject dogs from hospitalization to discharge, and the amount of physical activity and Sleeping/Resting time was measured. A video was captured using a camera to confirm the subjects’ movement from 22:00–6:00 the next day; the light was turned off at night, and their activity was recorded with an infrared camera (TS-WPTCAM, I-O DATA, Ishikawa, Japan). The dogs were hospitalized in an intensive care unit (ICU-MENIOS; Menix, Saitama, Japan). The size of the room was 580 × 750 × 650 mm or 1160 × 750 × 650 mm, such that the animals could stand and walk, depending on their body size. We prepared a larger cage for a large patient, but in this experiment, a larger cage was not necessary. Water was provided in these cages for *ad libitum* drinking.

**Table 1:** Comparison of PLUS CYCLE^®^ and video observations of hospitalized dogs.

No.	Age (y)	BW (kg)	Gender	Breeds	Operation
1	10	5.3	Unspayed female	Toy Poodle	PEA-CTR-IOL (OD) ISP (OS)
2	7.5	4.6	Spayed female	Toy Poodle	PEA-IOL (OD)
3	9.9	11	Unnutered male	Shiba Inu	Ahmed glaucoma valve implantation (OS)
4	13.3	9.5	Spayed female	Mix breeds	ISP (OU)
5	6.9	10.3	Unspayed female	Shiba Inu	ISP (OD)
6	5.5	4.6	Spayed female	Miniature Pincher	PEA-IOL
7	13.9	4.7	Unneutered male	Miniature Dachshund	PEA-IOL (OS)
8	6.9	6.8	Neutered male	Bichon Frise	PEA-IOL after superficial keratectomy of corneal degeneration, transpupil laser retinopexy (OD)
9	9.9	2.7	Spayed female	Toy Poodle	PEA-IOL (OU)

This is an overview of the nine dogs recruited for Experiment 1. Abbreviations: ISP, evisceration and implantation of intraocular silicone prosthesis; PEA-IOL, phacoemulsification and aspiration with implantation of intraocular lens; PEA-CTR-IOL, phacoemulsification and aspiration with implantation of capsular tension ring and intraocular lens.

The times of head-raising, standing, and walking movements were recorded from the video, and the duration of each movement was then calculated. Downloaded activity data from PLUS CYCLE^®^ were then compared with the video analysis data. The correlation between the duration of each movement in the video and the duration recorded by PLUS CYCLE^®^ data was evaluated. For the re-evaluation of false-positive detections, each detected activity signal was cross-checked against video footage. Activity events were categorized as “body repositioning,” “jerking,” or “no observable movement” (i.e., false positives where the PLUS CYCLE^®^ device detected a signal but no visible movement was confirmed in the video). The total number of false-positive signals was calculated as the sum of “no observable movement” events across all monitored periods.

The time when no movement was observed in the video was defined as “inactive time,” and it was compared hourly to see if it was equal to the “Sleeping/Resting time” (the time when no movement was recorded by PLUS CYCLE^®^). Sleeping/Resting time is when the accelerometer does not detect the movement of the animal to which it is attached, and this study cannot confirm that the animal was sleeping at that time.

### Experiment 2: Detection of sleep disturbances by PLUS CYCLE^®^

Six dogs that underwent phacoemulsification and aspiration (PEA) surgery for ophthalmic cataracts at the Veterinary Education Hospital of the Okayama University of Science were recruited for Experiment 2. The mean age and body weight were 7.2 ± 3.7 years (range, 1.75–13.17 years) and 5.5 ± 2.1 kg (range, 2.3–8.1 kg), respectively. There was one unneutered male, one unspayed female, one neutered male, and three spayed females. The breeds included Toy Poodle (n = 2), Miniature Dax (n = 1), Miniature Pinscher (n = 1), Bichon Frise (n = 1) and American Cocker Spaniel (n = 1). In this study, all dogs were non-brachycephalic, which excluded the influence of anatomical predisposition to sleep-related breathing disorders such as obstructive sleep apnea. Dogs with obvious pain prior to surgery by an ophthalmologist were excluded from the study. Acute Pain Scale (APS) scores were 0 (n = 3) and 1 (n = 3). We divided the cases into APS = 0 and APS = 1 groups (Table 2).

**Table 2:** A summary of the dogs used to detect sleep disturbances with PLUS CYCLE^®^.

No.	Age (y)	BW (kg)	Gender	Breeds	APS
1	8.2	2.3	Spayed female	Toy Poodle	0
2	1.8	8.1	Unspayed female	American Cocker Spaniel	0
3	7.4	4.6	Spayed female	Toy Poodle	0
4	13.2	6.7	Unneutered male	Miniature Dachshund	1
5	5.5	4.6	Spayed female	Miniature Pincher	1
6	6.9	6.8	Neutered male	Bichon Frise	1

This list shows the patients recruited in Experiment 2. Abbreviations: APS, Acute Pain Scale.

A PLUS CYCLE^®^ device was attached to the dogs during hospitalization, from the postoperative period to discharge. The anesthetic protocol for PEA surgery was as follows: dogs were intravenously (IV) administered fentanyl (3 μg/kg), ketamine (0.5 mg/kg), and atropine (15 μg/kg) for premedication. Anesthesia was induced with alfaxalone (administered IV to effect) and maintained with sevoflurane in 100% oxygen and constant rate infusions of fentanyl (5–10 μg/kg/h) and ketamine (0.6 mg/kg/h). Dogs were intubated and mechanically ventilated to maintain normocapnia. Rocuronium was administered IV for an initial loading dose (0.1 mg/kg) followed by an infusion at 0.2 mg/kg/h. After surgery, all drug administrations were discontinued, and the dogs were allowed to recover from anesthesia. During the recovery period, dogs received IV administration of methylprednisolone sodium succinate (5 mg/kg) over 30 min for anti-inflammation. After confirming recovery of consciousness and heads-up, the dogs were returned to the hospital room. Subsequently, anesthesiologists assessed postoperative pain using the Colorado State University APS. The APS scores were as follows: APS = 0, comfortable/no pain; 1, mild pain; 2, mild to moderate pain; and 3, moderate pain; 4, severe pain. The details of the APS criteria are based on the literature [14]. The pre- and postoperative “Sleeping/Resting time” and the amount of physical activity recorded throughout the night (22:00–6:00) by PLUS CYCLE^®^ from the day before surgery until 3 days (pre, post 1, and post 2) were compared.

### Statistical analysis

All data are presented as the mean ± standard deviation. Statistical analyses were performed using a data analysis software package (GraphPad Prism version 9.5.0 for macOS; GraphPad Software Inc., San Diego, CA, USA). Pearson’s correlation coefficients were calculated to assess the correlation between the amount of physical activity of PLUS CYCLE^®^ and the active, standing, and walking times recorded by video. The relationship between the Sleeping/Resting time of the PLUS CYCLE^®^ and the inactive time of the video was determined using Pearson’s correlation coefficients. Normality of the data distribution was assessed using the Shapiro–Wilk test. In Experiment 1, neither the postoperative values of Sleeping/Resting time nor the amount of physical activity were normally distributed. Therefore, the Wilcoxon signed-rank test was used for paired comparisons. In Experiment 2, we also compared the variation in Sleeping/Resting time and the amount of physical activity before and after PEA by a one-way repeated measures analysis of variance (Dunnett’s test).

## Results

### Experiment 1

**Fig 1:**
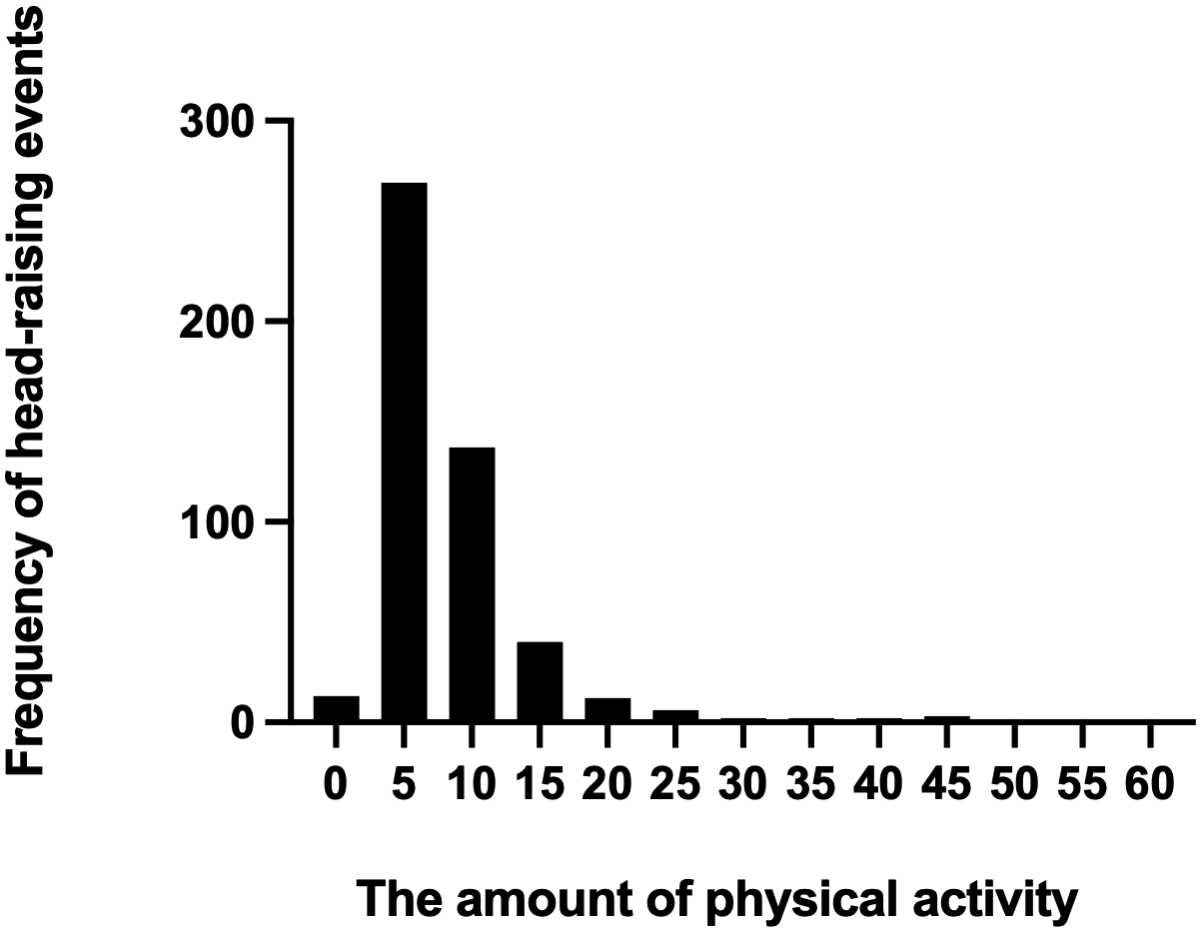
Distribution of the amount of physical activity recorded during head-raising movements. The histogram shows the frequency of the amount of physical activity measured by the PLUS CYCLE^®^ during manually confirmed head-raising events. This distribution was used to evaluate the detection range and consistency of the device.

During hospitalization, the dogs exhibited various movements at night, including head-raising, standing, and walking. The video recordings demonstrated that dogs were not always asleep at night. Daily activity data were summarized in 1-hour bins (see Supporting Information S1 File in S1 Data). By matching the recorded images with the numerical values obtained from the PLUS CYCLE^®^, we determined that the PLUS CYCLE^®^ was able to detect very slight movements, such as raising the head for a few seconds. Fig 1 shows a histogram of the amount of physical activity recorded by the PLUS CYCLE^®^ when the head was raised, indicating that the PLUS CYCLE^®^ was able to detect even the slightest movement, although the values varied depending on the size and speed of the head raising gesture. To assess the consistency of the accelerometer’s response to defined minimal movement (head-raising), we analyzed the distribution of record activity values corresponding to each head-raising event observed in video. The resulting histogram reflects the signal distribution pattern for this behavior.

**Fig 2:**
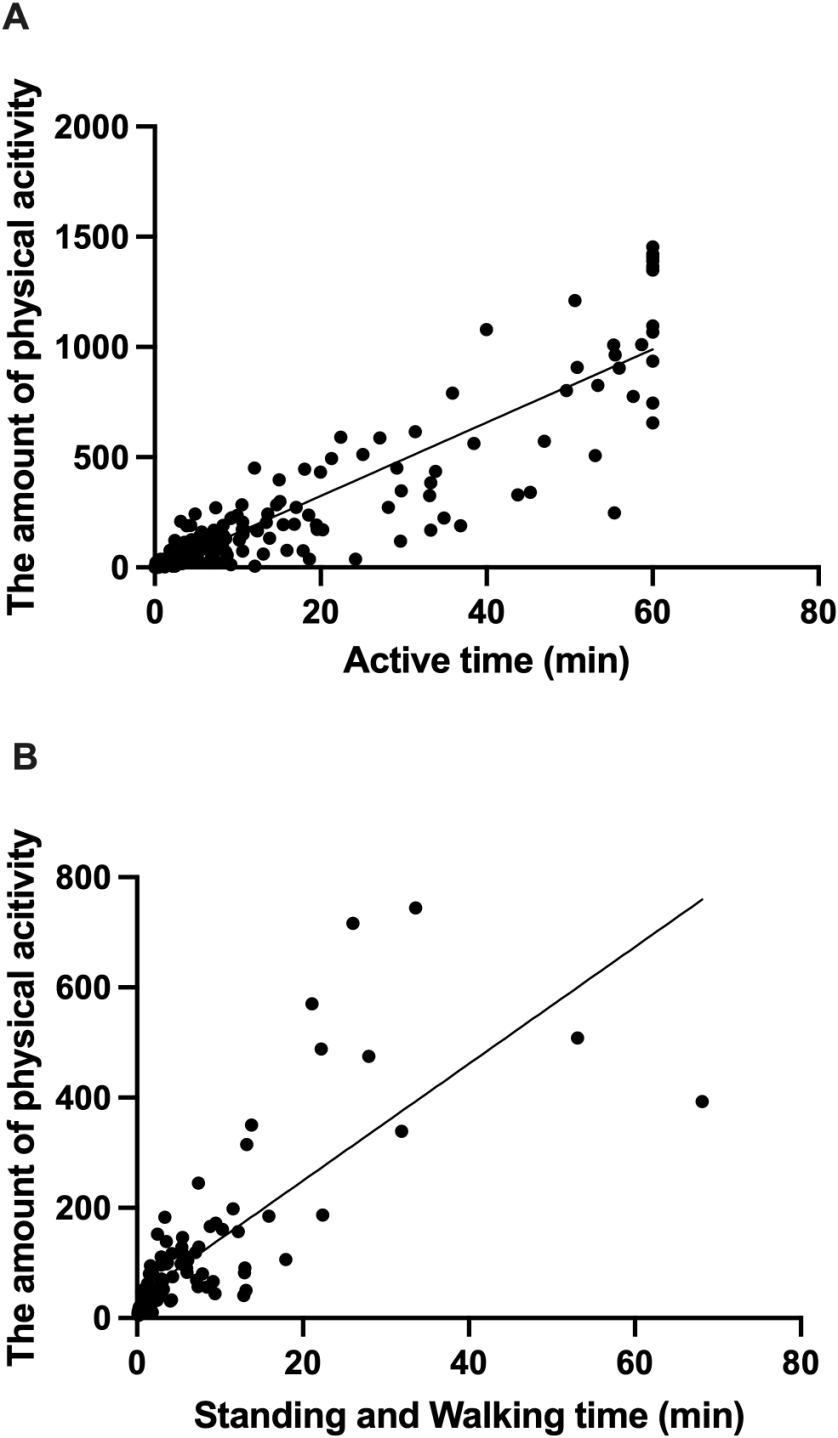
Correlation between the amount of physical activity recorded PLUS CYCLE^®^ and video-observed activity. (A) Correlation between the amount of physical activity measured by the PLUS CYCLE^®^ and total time of observable activity confirmed by video. (B) Correlation between the amount of physical activity and time spent standing and walking based on video observation.

**Fig 3:**
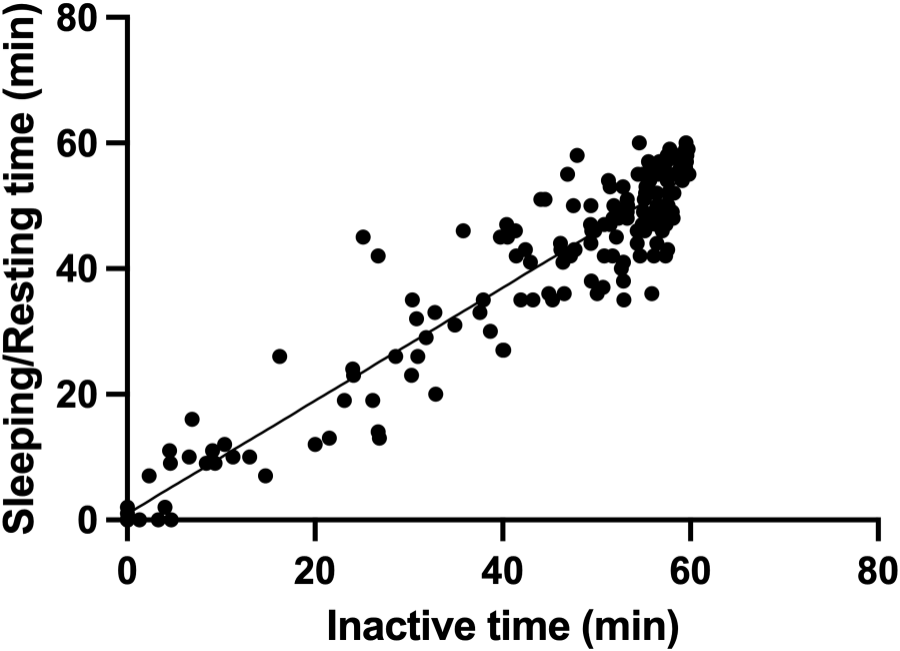
Correlation between Sleeping/Resting time recorded by PLUS CYCLE^®^ and inactive time observed by video. This figure shows the relationship between non-movement periods confirmed by video recordings and the corresponding Sleeping/Resting time recorded by the PLUS CYCLE^®^ device.

Data were collected using PLUS CYCLE^®^ and a video for 10,560 min. We determined the correlation between total hours of physical activity, Sleeping/Resting time, and inactive time. Statistical results for Fig 2A and 2B are provided in Supporting Information (S2 File in S1 Data). There was a significant positive correlation between the amount of physical activity in PLUS CYCLE^®^ and its duration, as confirmed by the video (Fig 2A) (p < 0.0001, r = 0.89). We observed a significant positive correlation between the duration of standing and walking movements, and the amount of physical activity during that period (Fig 2B) (p < 0.0001, r = 0.78). Details of the regression analysis in Fig 3 are included in Supporting Information (S3 File in S1 Data). There was a strong correlation between the inactive time observed in the videos and the Sleeping/Resting time in PLUS CYCLE^®^ (Fig 3) (p < 0.0001, r = 0.95). These tests confirm that PLUS CYCLE^®^ is suitable for measuring the activity and sleep status of dogs in hospitalized cages.

**Fig 4:**
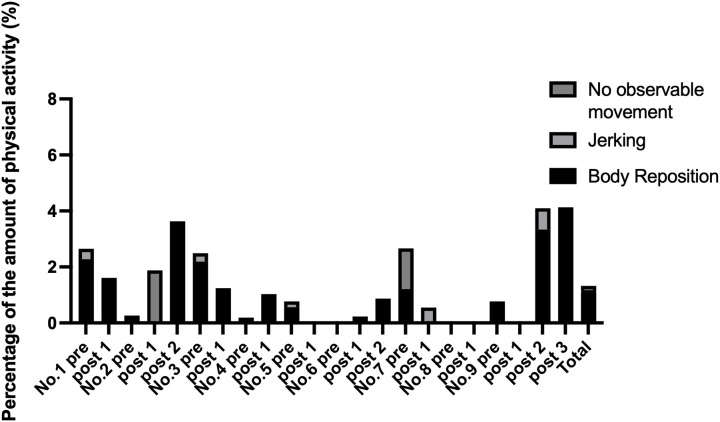
Verification of PLUS CYCLE^®^ signals during presumed sleep periods. The graph shows the percentage of PLUS CYCLE^®^ signals during presumed sleep periods. False positives were negligible, averaging less than 1% per case.

Upon re-evaluation of the activity signs recorded during the presumed sleep periods, we cross-checked video recording and PLUS CYCLE^®^ data to identify periods in which activity signals were recorded despite no visible movement being observed (Fig 4). Across all cases, the mean false-positive rate was approximately 1%, with the maximum rate observed being under 4% (see S4 and S5 Files in S1 Data for detailed analysis). The majority of these signals were found to correspond with subtle observable movements such as head-raising or body repositioning. Unmatched signals were rare and likely attributable to minor undetectable movements or video limitations such as blind spots or low contrast in dark-coated dogs. These findings suggest that potential false positives from the accelerometer are minimal and do not substantially affect the overall sleep assessment.

**Fig 5:**
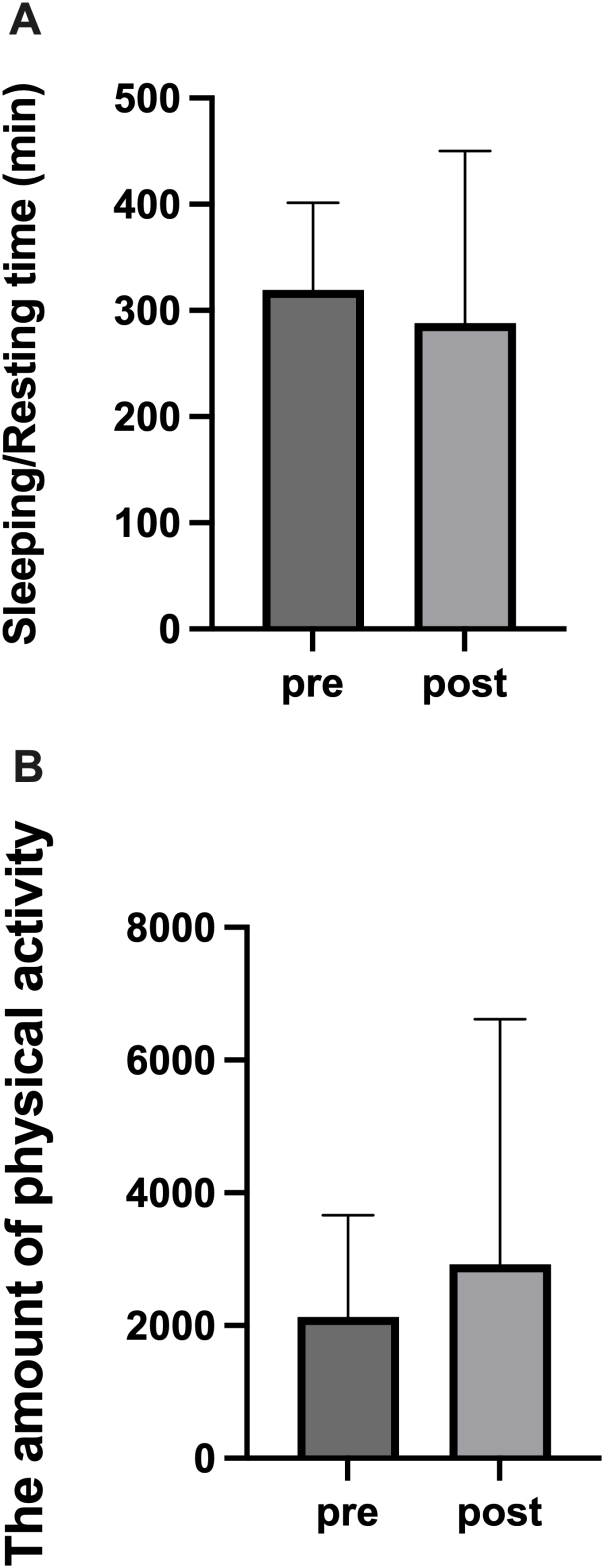
Comparison of sleep and activity the night before and after surgery in Experiment 1. (A) Sleeping/Resting time. (B) The amount of physical activity. Normality tests for data in Fig 5 are included in Supporting Information (S7–S10 Files in S1 Data). No statistically significant differences were observed. Bars represent mean ± SD.

In Experiment 1, we compared Sleeping/Resting time and the amount of physical activity collected by PLUS CYCLE^®^ on the night before surgery and the night of surgery (Fig 5). Statistical comparisons for APS groups in Fig 5 are shown in Supporting Information (S6 File in S1 Data). No statistically significant differences were observed in Sleeping/Resting time or the amount of physical activity between the night before and after surgery. However, a large individual variability was noted in the postoperative data, and several cases showed a tendency toward reduced rest time and increased activity levels.

Based on these findings, we hypothesized that differences in surgical procedures and preoperative pain might have contributed to the variability. Therefore, in Experiment 2, we standardized the surgical procedure (phacoemulsification and aspiration) and excluded cases with preoperative pain. This allowed us to evaluate the effect of postoperative acute pain, as assessed by the APS score, on rest-activity patterns under more uniform clinical conditions.

### Experiment 2

**Fig 6:**
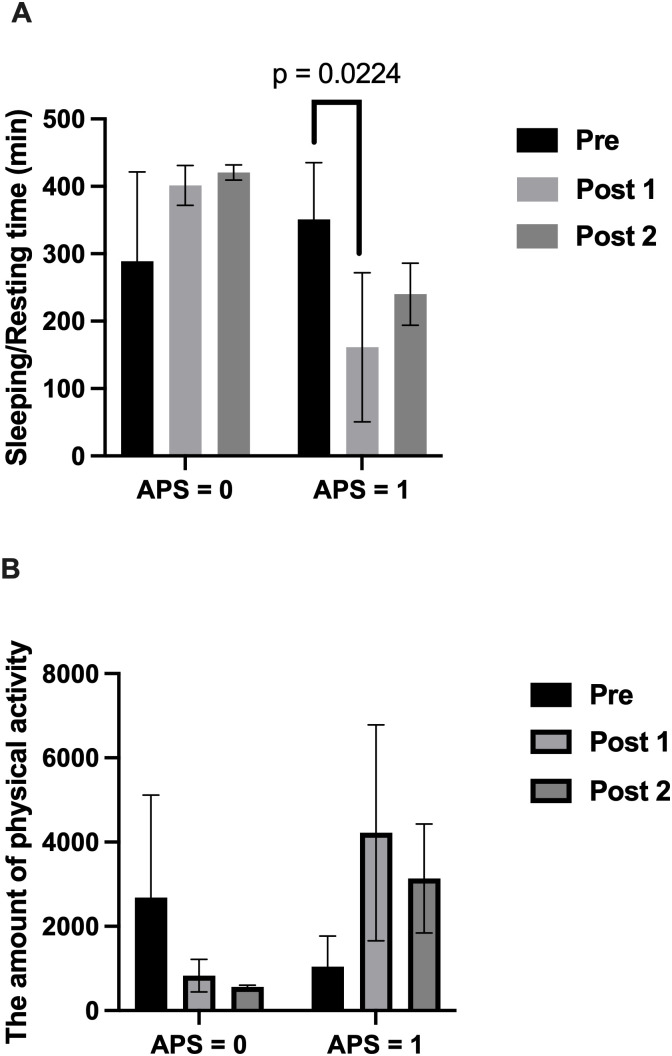
Comparison of postoperative rest and activity between APS score groups in Experiment 2. (A) Sleeping/Resting time in dogs with APS = 0 (n = 3) and APS = 1 (n = 3) groups. (B) The amount of physical activity in the same groups. Normality testing results for Fig 6 variables are provided in Supporting Information (S13–S24 Files in S1 Data). A significant difference was observed in Sleeping/Resting time (p = 0.0224). Bars represent mean ± SD. APS, Acute Pain Scale.

Hourly activity records for Experiment 2 are provided in Supporting Information (S11 File in S1 Data). Despite no postoperative pain or obvious postoperative complications, the anesthesiologist assessed the APS values, which varied based on a combination of factors from the patient’s condition. Full statistical output for Fig 6A and 6B is available in Supporting Information (S12 File in S1 Data). Dunnett’s multiple comparisons showed a significant decrease in the APS = 1 group at the first postoperative time (Fig 6A) Postoperative Sleeping/Resting time did not change significantly after PEA in the APS = 0 group compared with the preoperative day. On the second postoperative day, there was no decrease in Sleeping/Resting time in either group compared with that on the postoperative day. The amount of physical activity tended to decrease postoperatively in the APS = 0 group and increase postoperatively in the APS = 1 group compared with preoperatively (Fig 6B)

## Discussion

This study demonstrates that the PLUS CYCLE^®^ system is a feasible and non-invasive tool for assessing postoperative rest and activity in hospitalized dogs. By monitoring subtle movements and classifying periods of inactivity, the system provided insights into potential sleep disturbances associated with postoperative pain and discomfort. This approach may serve as an alternative to PSG in clinical veterinary settings.

In this study, we selected patients who underwent ophthalmologic surgery to evaluate postoperative sleep disturbances because we believed that postoperative body movements would not be restricted. For example, patients who undergo laparotomy may be less likely to stand up and walk owing to postoperative pain from the surgical wound. We could not confirm that the patients rubbed their eyes to complain of eye pain in the video, as would be recorded in humans.

In Experiment 1, the amount of physical activity recorded by PLUS CYCLE^®^ was positively correlated with the active time recorded by the video. The standing and walking time and the amount of physical activity recorded by PLUS CYCLE^®^ were confirmed by video. Therefore, the high summation of physical activities identified by PLUS CYCLE^®^ indicated that standing and walking were sustained for a long duration, preventing the dogs from resting. The inactive time recorded by the video and the Sleeping/Resting time measured by PLUS CYCLE^®^ were strongly correlated, thereby indicating the possibility of using PLUS CYCLE^®^ to accurately determine the resting state of dogs. Although no statistically significant differences were observed in Experiment 1, the variability in postoperative rest-activity data suggested that individual differences, preoperative pain, and variations in surgical procedure may have influenced the results. This consideration led us to conduct Experiment 2 under more controlled clinical conditions.

Although PSG provides the most comprehensive data on sleep stages and sleep architecture, its use in veterinary clinical settings is limited due to the need for electrodes, extended monitoring time, and the potential for restraint-induced stress. Furthermore, such procedures may not be feasible in postoperative patients, especially those recovering from ophthalmic surgery where undue stress or physical restraint could exacerbate discomfort or interfere with recovery. Thus, the use of a non-invasive device like PLUS CYCLE^®^ offers a practical alternative for assessing rest-activity patterns in hospitalized dogs.

In Experiment 1, despite evaluating postoperative changes in physical activity and resting time, no statistically significant differences were observed. One possible explanation is the heterogeneity of surgical procedures and postoperative pain levels, which may have masked meaningful changes in behavior. Therefore, in Experiment 2, we standardized the surgical procedure and stratified dogs based on postoperative pain scores (APS). This refinement revealed significant differences, suggesting that pain level is a key factor influencing postoperative behavior and rest patterns.

In Experiment 2, the Sleeping/Resting time and amount of physical activity on the day before hospitalization were used as controls to evaluate postoperative sleep disturbances. However, this differs from the patient’s daily sleep condition because of the environmental stress caused by hospitalization. In the APS = 0 group, the Sleeping/Resting time gradually increased and the amount of physical activity decreased, whereas in the APS = 1 group, the Sleeping/Resting time decreased and the amount of physical activity increased on postoperative day 1.

In Experiment 2, the observed decrease in sleep duration in the APS = 1 group suggests that postoperative discomfort may have contributed to restlessness, consistent with findings in human postoperative studies [15,16]. However, the lack of a significant decrease on postoperative day 2 may indicate a period of adaptation or the resolution of acute discomfort. Further studies are needed to assess whether these sleep disturbances are directly related to pain or other factors, such as environmental stress or medication effects. While no significant differences in sleep duration were detected between the APS = 0 and APS = 1 groups overall, the trend toward reduced sleep in the APS = 1 group aligns with previous findings that pain can disrupt sleep in postoperative patients. Further studies are warranted to assess the impact of analgesic protocols on sleep quality in this context.

Studies using activity monitors have been conducted in both dogs and humans. However, one important question is how well activity monitors can detect movement. Although various activity monitors have been used in animals to study activity [17–20], no studies have used an activity monitor to assess sleep in dogs. Previous studies using PLUS CYCLE^®^ have shown that it can objectively and accurately assess cat sleep [13]. This finding suggests that activity monitors can provide objective measures of rest periods, though further studies using PSG are necessary to confirm actual sleep states. These prompted us to evaluate the relationship between video-confirmed inactive and recorded sleep parameters. Several studies have classified behaviors from videos based on this method, and their criteria rate the state in which the eyes are closed for 2 min as being asleep [21]. However, in this study, it was not possible to confirm the eye condition at all times. Therefore, we evaluated sleep disturbance by assuming that the patients were not sleeping when they showed body movements. Drowsiness, a transitional state between wakefulness and sleep, can present with open or closed eyes, complicating visual confirmation of sleep states. Additionally, brief involuntary movements such as sleep jerks during REM sleep may have been missed due to the resolution limitations and lack of EEG monitoring. While our approach assumes that visible movement indicates wakefulness, we recognize that some brief or subtle behaviors may have been misclassified. Nonetheless, the consistency between video observations and accelerometer output supports the general reliability of the PLUS CYCLE^®^ system for detecting rest disturbances.

In human medicine, sleep disturbances are increasingly recognized as indicators of poor postoperative recovery and overall well-being [15]. Similarly, in veterinary settings, sleep assessment may serve as a potential welfare indicator, especially in postoperative care. However, further research is necessary to determine the extent to which increased activity during expected rest periods correlates with pain or discomfort in animals. To the best of our knowledge, this study is the first to report that postoperative sleep disturbances can occur in dogs, as observed in humans. In humans, postoperative sleep disturbance is thought to be related to various factors [15], including pain [16]. Although it has been suggested that the stress of hospitalization in veterinary medicine may result in sleep disturbances [8], no reports have directly addressed this. The results of the present study suggest that postoperative APS is a factor that causes sleep disturbance and may be related to postoperative pain. In human medicine [15], postoperative sleep disturbance is influenced not only by pain but also by the effects of opioid analgesics, which in some cases may lead to sleep disruption such as insomnia [16]. In our study, while dogs with APS = 1 received standard postoperative analgesia, the possibility remains that either mild residual pain or opioid-related effects may have contributed to the decreased rest time observed.

Furthermore, the sleeping/resting time measured by the PLUS CYCLE^®^ system showed a high correspondence with periods in which no movement was observed on video recordings and the device recorded zero activity counts. Although subtle movements such as minor twitches or brief postural adjustments might escape detection due to the device’s 1-minute resolution, we selectively analyzed only clearly distinguishable head-raising events preceded and followed by inactivity. This ensured the reliability of our behavioral labeling and minimized potential confounding.

The consistency between inactivity on video and zero-count periods strongly supports the validity of the sleeping/resting time parameter as a proxy for sleep or rest. The additional finding that these values correlate with postoperative APS scores further suggests that the metric reflects clinically relevant behavioral changes.

This study has several limitations. First, the small sample size may limit the generalizability of the findings. Second, technical limitations such as reduced visibility in dark-coated dogs and blind spots in infrared video recordings may have affected the detection of subtle movements. Third, while PSG remains the gold standard for sleep assessment, its application in clinical veterinary settings is limited due to the need for electrodes and extended monitoring periods, which may interfere with natural sleep patterns. Therefore, we employed the PLUS CYCLE^®^ system as a non-invasive alternative to estimate rest and sleep duration. Lastly, the 1-minute recording interval may not capture brief, subtle movements, potentially resulting in false positives or missed detections, as discussed in the analysis of false-positive data (see S4 and S5 Files in S1 Data).

## Conclusion

PLUS CYCLE^®^ accurately monitors the behavior of hospitalized dogs, including their resting state, and may monitor patients’ postoperative sleep status. This will enable the detection of previously unreported postoperative sleep disturbances and allow for optimal postoperative monitoring.

## Supporting information

S1 Data (DOCX)
