# Supplementary material for: Utility of PLUS CYCLE to measure physical activity and sleep duration and detect postoperative sleep disturbances in hospitalized dogs
Source: PLoS One. 2025 Jun 26;20(6):e0318475. doi: 10.1371/journal.pone.0318475 (PMC12200879; doi:10.1371/journal.pone.0318475)
Supplement: S1 Data — (DOCX) [file pone.0318475.s001.docx]

Supporting Information Legends

S1_ActivityData_Experiment1.xlsx

This Excel file contains hourly physical activity data, resting time, and video-recorded inactivity duration for 9 dogs (Case 1–9) in Experiment 1.

S2_StatisticalOutput_Exp1_Fig2.pdf

Statistical or normality analysis result for Exp1 Fig2.

S3_StatisticalOutput_Exp1_Fig3.pdf

Statistical or normality analysis result for Exp1 Fig3.

S4_FalsePositive_SummaryTable.xlsx

This Excel file summarizes the number of false-positive events detected by PLUS CYCLE in Experiment 1.

S5_FalsePositive_AnalysisDetails.xlsx

This Excel file presents a detailed breakdown of the percentage of false-positive events for each dog in Experiment 1.

S6_StasticalOutput_Fig5.pdf

Statistical or normality analysis result for Fig5.

S7_Normality_Fig5_SleepingRestingTime_Pre.pdf
Statistical or normality analysis result for Fig5 SleepingRestingTime Pre.

S8_Normality_Fig5_SleepingRestingTime_Post.pdf

Statistical or normality analysis result for Fig5 SleepingRestingTime Post.

S9_Normality_Fig5_PhysicalActivity_Pre.pdf

Statistical or normality analysis result for Fig5 PhysicalActivity Pre.

S10_Normality_Fig5_PhysicalActivity_Post.pdf

Statistical or normality analysis result for Fig5 PhysicalActivity Post.

S11_ActivityData_Exp2.xlsx

This file contains the sleeping/resting time and the amount of physical activity of dogs in Experiment 2 across three time periods: pre, post1, and post2.

S12_StasticalOutput_Exp2_Fig6.pdf

Statistical or normality analysis result for Exp2 Fig6.

S13_Normality_Fig6_SleepingRestingTime_APS0_Pre.pdf

Statistical or normality analysis result for Fig6 SleepingRestingTime APS0 Pre.

S14_Normality_Fig6_SleepingRestingTime_APS0_Post1.pdf

Statistical or normality analysis result for Fig6 SleepingRestingTime APS0 Post1.

S15_Normality_Fig6_SleepingRestingTime_APS0_Post2.pdf

Statistical or normality analysis result for Fig6 SleepingRestingTime APS0 Post2.

S16_Normality_Fig6_SleepingRestingTime_APS1_Pre.pdf

Statistical or normality analysis result for Fig6 SleepingRestingTime APS1 Pre.

S17_Normality_Fig6_SleepingRestingTime_APS1_Post1.pdf
Statistical or normality analysis result for Fig6 SleepingRestingTime APS1 Post1.

S18_Normality_Fig6_SleepingRestingTime_APS1_Post2.pdf

Statistical or normality analysis result for Fig6 SleepingRestingTime APS1 Post2.

S19_Normality_Fig6_PhysicalActivity_APS0_Pre.pdf

Statistical or normality analysis result for Fig6 PhysicalActivity APS0 Pre.

S20_Normality_Fig6_PhysicalActivity_APS0_Post1.pdf

Statistical or normality analysis result for Fig6 PhysicalActivity APS0 Post1.

S21_Normality_Fig6_PhysicalActivity_APS0_Post2.pdf

Statistical or normality analysis result for Fig6 PhysicalActivity APS0 Post2.

S22_Normality_Fig6_PhysicalActivity_APS1_Pre.pdf

Statistical or normality analysis result for Fig6 PhysicalActivity APS1 Pre.

S23_Normality_Fig6_PhysicalActivity_APS1_Post1.pdf

Statistical or normality analysis result for Fig6 PhysicalActivity APS1 Post1.

S24_Normality_Fig6_PhysicalActivity_APS1_Post2.pdf

Statistical or normality analysis result for Fig6 PhysicalActivity APS1 Post2.
